# Direct Medical Cost of Type 2 Diabetes in Singapore

**DOI:** 10.1371/journal.pone.0122795

**Published:** 2015-03-27

**Authors:** Charmaine Shuyu Ng, Matthias Paul Han Sim Toh, Yu Ko, Joyce Yu-Chia Lee

**Affiliations:** 1 Department of Pharmacy, Faculty of Science, National University of Singapore, Singapore, Singapore; 2 Information Management, Central Regional Health Office, National Healthcare Group, Singapore, Singapore; 3 Saw Swee Hock School of Public Health, National University of Singapore and National University Health System, Singapore, Singapore; 4 School of Pharmacy, College of Pharmacy, Taipei Medical University, Taipei, Taiwan; London School of Hygiene and Tropical Medicine, UNITED KINGDOM

## Abstract

Due to the chronic nature of diabetes along with their complications, they have been recognised as a major health issue, which results in significant economic burden. This study aims to estimate the direct medical cost associated with type 2 diabetes mellitus (T2DM) in Singapore in 2010 and to examine both the relationship between demographic and clinical state variables with the total estimated expenditure. The National Healthcare Group (NHG) Chronic Disease Management System (CDMS) database was used to identify patients with T2DM in the year 2010. DM-attributable costs estimated included hospitalisations, accident and emergency (A&E) room visits, outpatient physician visits, medications, laboratory tests and allied health services. All charges and unit costs were provided by the NHG. A total of 500 patients with DM were identified for the analyses. The mean annual direct medical cost was found to be $2,034, of which 61% was accounted for by inpatient services, 35% by outpatient services, and 4% by A&E services. Independent determinants of total costs were DM treatments such as the use of insulin only (p<0.001) and the combination of both oral medications and insulin (p=0.047) as well as having complications such as cerebrovascular disease (p<0.001), cardiovascular disease (p=0.002), peripheral vascular disease (p=0.001), and nephropathy (p=0.041). In this study, the cost of DM treatments and DM-related complications were found to be strong determinants of costs. This finding suggests an imperative need to address the economic burden associated with diabetes with urgency and to reorganise resources required to improve healthcare costs.

## Introduction

Globally, the total number of people with diabetes mellitus (DM) is projected to rise from 171 million in 2000 to 366 million in 2030 [1]. There is a growing epidemic of diabetes mellitus, type 2 in particular, in the Asia-Pacific region [2, 3]. According to current estimates, the DM population in this region is the largest in the world, with approximately 47.3 million, which is 46% of the global burden of this disease [4]. In Singapore, as in many developed countries, DM is a growing public health problem. The prevalence of DM has risen to 12.3% in 2013, from 8.2% in 2004 and 9% in 1998 [5–7], surpassing other Asian countries such as Hong Kong (9.5%), Japan (7.2%) and Taiwan (5.7%) [8]. Moreover, DM is the tenth leading cause of death in Singapore, accounting for 1.7% of total deaths in 2011 [9].

Diabetes is a chronic medical condition associated with numerous complications that makes it a substantial economic burden incurred by individuals, healthcare systems and society as a whole [10]. In 2007, the global health expenditure to treat and prevent DM and its complications was estimated to be at least US$232 billion [8]. Depending on available treatments and local prevalence, the direct costs of DM consume from 2.5% to 15.0% of annual healthcare budgets [11].

Despite the large number of people with DM, the financial burden in Singapore attributed to DM has not been investigated. Because type 2 diabetes mellitus (T2DM) accounts for approximately 90% of DM cases and its prevalence increases with ageing, understanding the patterns of resource use and cost associated with T2DM is becoming increasingly important for policymakers and budget planners. Therefore, this study aims to identify the total direct medical cost of T2DM in Singapore and to examine the relationship between direct medical costs and individual demographic characteristics, DM treatments (exercise or diet, taking oral medications only, taking insulin only and taking both insulin and oral medications), disease control, complications and comorbidities.

## Methods

### Study design

This study adopted a prevalence-based ‘epidemiological’ approach, employing a bottom-up methodology to estimate different cost components. The prevalence approach can yield more precise estimates because it ascertains the current economic burden of a disease rather than projected ones [12, 13]. The perspective for this study was that of the healthcare system (i.e., National Healthcare Group (NHG) institutions). This study was approved by the National Healthcare Group Domain Specific Review Board (NHG-DSRB).

### Data source

This was a cross-sectional study of T2DM patients who had received care in any of the NHG institutions in 2010. The NHG is public funded and provides inpatient and ambulatory care (primary care, specialist outpatient and 24-hour emergency) services through a network of 3 acute hospitals, 1 national center, 9 primary care clinics and 3 specialty institutes serving the population in the central and western parts of Singapore. The 9 primary care clinics, also known as polyclinics, had a service load of 3.7 million attendances in 2010, which accounted for 60% of all public sector primary care attendances [14]. Data was drawn from the NHG Chronic Disease Management System (CDMS), which serves as an operational disease registry within the NHG. The CDMS was commissioned in 2007 to enhance the delivery of care for patients with DM and to facilitate greater efficiency in outcome measurement. It links key clinical data of patients with DM across the NHG healthcare cluster, including records of visits to physicians, nurses, and allied health professionals, as well as medication and laboratory test records [15]. In addition, it also includes registration and financial cost data related to the care of chronic diseases.

### Patient selection

Patients with T2DM were identified using the International Classification of Diseases Ninth Revision (ICD-9-CM) with diagnostic code of 250 as primary or secondary diagnosis, or using pharmacy medication records or laboratory data in the CDMS. Diabetes complications and comorbidities were also identified using ICD-9-CM codes, while only DM-related medications and laboratory data were based on inpatient and outpatient encounters at the hospital or outpatient clinics that were registered with the CDMS. Systematic sampling was conducted for 98,592 identified DM patients (i.e., every 197^th^ patient was selected). Informed consent was not obtained from the patients as the data was de-identified prior to analysis.

This study included patients who satisfied at least one of the following three criteria: (1) assigned ICD-9-CM code of 250; (2) attended treatment for DM for 1 year in any NHG institution; or (3) prescribed any anti-diabetic medication. Patients with type 1 DM and women with gestational diabetes were excluded.

### Laboratory-derived measures related to DM

Measures for DM-related physical examinations were included and categorised as follow: (1) body mass index (BMI) (kg/m^2^): <18.50 = underweight; 18.50–24.99 = normal; >25.00 = overweight and obese [16], (2) glycated haemoglobin (HbA1c) (%): ≤7.0 = good disease control; 7.1–8.0 = sub-optimal disease control; >8.0 = poor disease control, (3) low-density lipoprotein cholesterol (LDL-c) (mmol/L): <2.6 = optimal; 2.6–4.0 = near optimal; >4.0 = high, (4) urine albumin-to-creatinine ratio (UACR) (albumin/24h): <30mg = normal; 30-299mg = microalbuminuria; >300mg = macroalbuminuria [17, 18].

### Estimation of costs

Direct DM-related costs were classified by the type of service, including inpatient hospitalisation, accident and emergency (A&E) and ambulatory outpatient care (physician visits, allied health visits, laboratory tests and medications). Allied health visits include foot screening, eye screening, dietary services and health education. The total medical costs were estimated by the total before-subsidy charges, which is the total medical bill before any deduction for government subsidies or insurance claims. All costs reported were in Singapore currency (S$) for year 2010 prices.

The cost of inpatient care and A&E services were estimated by the total charge based on the length of stay and resources used. Any A&E visits that resulted in hospitalisation were included as inpatient care costs. Unit costs used in the estimation of physician visits, which included visits to primary care clinics (polyclinics) and specialist outpatient clinics (hospitals), were equal to the standardised rate for physician visits at all NHG primary care clinics and hospitals. Therefore, costs were estimated by multiplying the number of physician visits by the unit cost of a visit. Unit costs for allied health visits, laboratory tests and medications were estimated via the same method as physician visits. The cost for drugs other than anti-diabetic medications was not included. Unit costs for all services rendered were provided by the NHG and are in Singapore dollars. Direct non-medical costs, such as transportation expenses and indirect costs were not included.

### Statistical methods

Healthcare cost data are often positively skewed because a relatively small proportion of patients incur extremely high costs [19, 20]. Such problems were dealt with by logarithmic transformation of the cost data [21]. Descriptive statistics (frequency, percentage, mean, median, standard deviation and 90^th^ percentile) were used for demographic information and expenditures. To identify the factors affecting total costs, a multiple linear regression model was developed to evaluate the relationship of both demographic and clinical state variables (HbA1c, DM treatments, complications and comorbidities) to the total calculated expenditure. All statistical analyses were performed using SPSS version 21.0 (SPSS Inc., Chicago, IL, USA).

## Results

### Patient characteristics

A total of 98,592 patients in the NHG CDMS (2010) were identified as patients with DM. After applying the selection criteria and a systematic sampling, 500 patients were included in the analyses. The socio-demographic profile of the patients is shown in Table 1. The patients were equally distributed between the two genders (55.4% female). The mean (±SD) age was 69.0 ± 9.4 years, and most study patients were Chinese (77.6%) and non-smokers (89.8%). Although a greater proportion of patients was overweight (42.6%), most had good disease control (44.6%), optimal LDL-c (43.2%) and normal UACR (41.2%). Of the 69.2% of DM patients who were on anti-diabetic medications, the majority used oral medications (57.2%), while only 3% were treated with insulin and the remaining 9% used both insulin and oral medications. Nephropathy (57.2%) and cardiovascular conditions (34.2%) were common DM complications among the cohort. The distributions of subgroups were similar between patients with at least one inpatient visit and those without any inpatient visit.

**Table 1:** Socio-demographic and clinical characteristics of patients with diabetes mellitus, CDMS 2010.

Characteristic	n (%)^a^ or mean ± standard deviation					
	Overall (n = 500)^b^		≥1 inpatient visit (n = 83)^c^		0 inpatient visit (n = 417)^d^	
***Individual level factors***						
**Age** (years)	69.0	±9.4	71.7	±9.5	68.8	±9.3
**Gender**						
Female	277	(55.4)	45	(54.2)	185	(55.6)
Male	223	(44.6)	38	(45.8)	232	(44.4)
**Race**						
Chinese	388	(77.6)	61	(73.5)	327	(78.4)
Malay	57	(11.4)	10	(12.0)	47	(11.3)
Indian	34	(6.8)	6	(7.2)	28	(6.7)
Others	21	(4.2)	6	(7.2)	15	(3.6)
**Smoking status**						
Non-smoker	449	(89.8)	75	(90.4)	374	(89.7)
Smoker	51	(10.2)	8	(9.6)	43	(10.3)
***Physical examination***						
**BMI (kg/m** ^**2**^ **)** (n = 378)^1^ (n = 45)^2^ (n = 333)^3^	26.1	±4.7	26.0	±5.0	26.1	±4.6
Underweight	7	(1.4)	2	(2.4)	5	(1.2)
Normal	158	(31.6)	16	(19.3)	142	(34.1)
Overweight	213	(42.6)	27	(32.5)	186	(44.6)
**Blood pressure (mmHg)** (n = 414)^1^ (n = 56)^2^ (n = 358)^3^						
Systolic	132.2	±14.0	134.9	±17.1	131.8	±13.4
Diastolic	70.4	±7.5	71.2	±9.3	70.3	±7.2
**HbA1c (%)** (n = 441)^1^ (n = 73)^2^ (n = 368)^3^	7.3	±1.2	7.3	±1.5	7.3	±1.2
Good disease control	223	(44.6)	37	(44.6)	186	(44.6)
Sub-optimal disease control	134	(26.8)	19	(22.9)	115	(27.6)
Poor disease control	84	(16.8)	17	(20.5)	67	(16.1)
**LDL-c level (mmol/L)** (n = 398)^1^ (n = 61)^2^ (n = 337)^3^	2.6	±0.8	2.7	±1.1	2.6	±0.7
Optimal	216	(43.2)	35	(42.2)	181	(43.4)
Near optimal	169	(33.8)	23	(27.7)	146	(35.0)
High	13	(2.6)	3	(3.6)	10	(2.4)
**Serum creatinine (μmol/L)** (n = 424)^1^ (n = 81)^2^ (n = 343)^3^	102.1	±87.6	137.0	±145.4	93.9	±64.8
**UACR** (n = 321)^1^ (n = 39)^2^ (n = 282)^3^						
Normal	206	(41.2)	15	(18.1)	191	(45.8)
Microalbuminuria	94	(18.8)	20	(24.1)	74	(17.7)
Macroalbuminuria	21	(4.2)	4	(4.8)	17	(4.1)
***Diabetes treatment***						
Diet or exercise only	154	(30.8)	27	(32.5)	127	(30.5)
Oral anti-diabetic medication only	286	(57.2)	42	(50.6)	244	(58.5)
Insulin only	15	(3.0)	8	(9.6)	7	(1.7)
Oral and insulin	45	(9.0)	6	(7.2)	39	(9.4)
***Diabetes complications***						
Nephropathy	286	(57.2)	63	(75.9)	223	(53.5)
Cardiovascular	171	(34.2)	49	(59.0)	122	(29.3)
Retinopathy	75	(15.0)	24	(28.9)	51	(12.2)
Peripheral vascular disease	73	(14.6)	27	(32.5)	46	(11.0)
Cerebrovascular	71	(14.2)	32	(38.6)	39	(9.4)
***Diabetes comorbidity***						
Dyslipidaemia	483	(96.6)	77	(92.8)	406	(97.4)
Hypertension	441	(88.2)	81	(97.6)	360	(86.3)

^a^ Percentages may not add up to 100% due to missing values

^b^ n = 500 otherwise stated in the brackets

^c^ n = 83 otherwise stated in the brackets

^d^ n = 417 otherwise stated in the brackets

BMI = body mass index, HbA1c = glycated haemoglobin, LDL-c = low-density lipoprotein cholesterol, UACR = urine albumin-to-creatinine ratio

### Annual costs of diabetes

**Fig 1:**
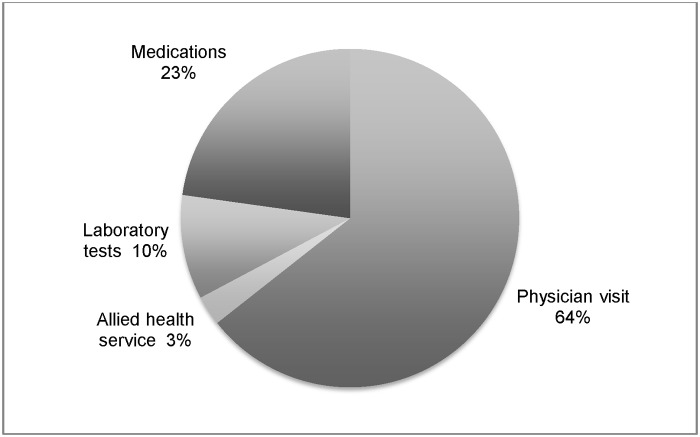
Components of outpatient costs.

The mean annual direct cost was S$2,034.6 (US$1.0 = S$1.3 as of 31 December 2010) [22], of which S$1,237.2 accounted for by inpatient services, S$84.2 by A&E services and S$713.2 by outpatient services (Table 2). Of the total healthcare expenditure, the main cost driver was inpatient costs (60.8%), while A&E services (4.1%) were only a small portion of the total costs. The major source of costs for outpatient services was physician visits, which accounted for 22.6% of the total healthcare expenditure and 64.0% of total outpatient expenditure (Fig. 1).

**Table 2:** Direct medical costs of diabetes mellitus paid by the hospital.

Costs variables	Total (S$)		%^a^	Mean	SD	Median	90th percentile
*Overall (n = 500)*							
**Inpatient costs**		**618,622.0**	**60.8**	1,237.2	± 4,085.8	0.0	2,846.4
**Accident & Emergency**		**42,084.4**	**4.1**	84.2	± 277.6	0.0	300.4
**Outpatient costs**		**356,600.8**	**35.1**				
Physician visit	229,506.0		22.6	459.0	± 396.8	325.0	974.0
Allied health service	10,061.0		1.0	20.1	± 30.0	19.6	40.8
Laboratory tests	35,990.0		3.5	72.0	± 52.5	68.9	114.3
Medications	81,043.9		8.0	162.1	± 220.4	101.0	377.5
**Total**		**1,017,306.2**		**2,034.6**	**± 4,351.0**	**664.1**	**4,209.6**
***≥ 1 inpatient visit (n = 83)***							
**Inpatient costs**		**618,621.0**	**84.8**	7,453.3	± 7,395.5	3,740.5	16,858.9
**Accident & Emergency**		**33,745.6**	**4.6**	406.6	± 550.2	301.2	722.4
**Outpatient costs**		**77,021.4**	**10.6**				
Physician visit	57,851.0		7.9	697.0	± 479.0	612.0	1,344.4
Allied health service	2,022.9		0.3	24.4	± 50.0	0.0	52.3
Laboratory tests	5,523.2		0.8	66.5	± 63.3	58.2	154.5
Medications	11,624.3		1.6	140.1	± 232.2	62.0	302.4
**Total**		**729,388.0**		**8,787.8**	**± 7,660.1**	**5,160.4**	**18,322.6**
***0 inpatient visit (n = 417)***							
**Accident & Emergency**		**8,338.8**	**2.9**	20.0	± 88.9	0.0	0.0
**Outpatient costs**		**279,579.4**	**97.1**				
Physician visit	171,655.0		59.6	411.6	± 360.7	296.0	834.2
Allied health service	8,038.0		2.8	19.3	± 24.1	19.6	35.4
Laboratory tests	30,466.7		10.6	73.1	± 50.1	70.9	108.2
Medications	69,419.6		24.1	166.5	± 218.0	114.7	386.7
**Total**		**287,918.2**		**690.5**	**± 481.3**	**588.2**	**1,200.6**

^a^ Percentages may not add up to 100% due to missing values

Patients with at least one inpatient admission had higher mean total costs (S$8,787.8) than those who had no inpatient admission (S$690.5), with the bulk of costs resulting from inpatient services (S$7,453.3). Conversely, patients with no inpatient visits had a substantially higher proportion of overall outpatient costs.

### Factors affecting the total costs

Using multiple linear regression with log transformation, the total cost of DM was significantly associated with DM treatments (taking insulin only or both oral medications and insulin) and DM-related complications (cerebrovascular, cardiovascular, and peripheral vascular diseases and nephropathy). This model explained 23.0% of the variance in costs (Table 3). Age, gender, race, smoking status, disease control, taking only oral medication, having retinopathy and comorbidities were not independently associated with cost. The combination of oral medications and insulin resulted in an average increment in annual total cost (17.5%, p = 0.047), while the use of only insulin led to a higher increment (53.2%, p<0.001) when compared with patients who were only on dietary control and healthy lifestyle advice alone. Taking the absence of complications as reference, the cost of DM was higher when complications were present except in the case of retinopathy.

**Table 3:** Factors influencing the total annual cost of diabetes (n = 500)^a^.

Characteristic	β-coefficient	95% CI		P value
***Individual level factors***				
**Age** (per year increase)	0.000	-0.004	0.005	0.900
**Gender**				
Male (reference)				
Female	0.034	-0.047	0.114	0.412
**Race**				
Chinese (reference)	0.000			
Malay	0.102	-0.021	0.225	0.104
Indian	0.039	-0.120	0.198	0.632
Others	0.143	-0.050	0.336	0.146
**Smoking status**				
Non-smoker (reference)				
Smoker	-0.043	-0.178	0.091	0.528
***Physical examination***				
**HbA1c (%)** (n = 441)				
Good disease control (reference)				
Sub-optimal disease control	0.001	-0.097	0.098	0.989
Poor disease control	0.066	-0.055	0.187	0.286
***Diabetes treatment***				
Diet or exercise only (reference)				
Oral medication only	0.079	-0.024	0.182	0.132
Insulin only	0.532	0.276	0.788	<0.001
Oral and insulin	0.175	0.002	0.348	0.047
***Diabetes complications*** *(Absent reference)*				
Cerebrovascular	0.310	0.189	0.430	<0.001
Cardiovascular	0.150	0.054	0.245	0.002
Peripheral vascular disease	0.207	0.088	0.325	0.001
Nephropathy	0.123	0.005	0.240	0.041
Retinopathy	0.046	-0.070	0.161	0.436
***Diabetes comorbidity***				
None (reference)				
Either hypertension or dyslipidaemia	0.114	-0.101	0.328	0.298
Both hypertension and dyslipidaemia	0.071	-0.077	0.219	0.348

^a^ n = 500 otherwise stated in the brackets

HbA1c = glycated haemoglobin

## Discussion

This prevalence-based cost-of-illness study involved a large captive population with T2DM in Singapore. The analysis was based on cost and administrative data retrieved from the NHG disease registry for 2010. This is the first study to provide estimates of costs associated with diabetes care in Singapore.

The cost per patient estimate in this present study was S$2,034.6 (US$1,575.6), and this appears to be higher than the costs reported in other Asian countries. A study in India reported an estimate of US$525.5 per patient [23], while a study in China reported costs of US$1,501.7 per patient [11] for the management of DM. However, the costs reported in these studies were presented without accounting for inflation or difference between currency. Notably, hospital costs reported in the American and European continents were much higher than those obtained in this study [24–26]. Despite the cost differences, inpatient costs still remained the main cost driver of the total estimated expenditure, which was also noted in the earlier DM COI studies [25, 27–29]. Although the length of stay (LOS) was not reported in this study, the high cost of inpatient services were often strongly correlated to LOS [30, 31], with higher LOS resulting in higher costs. This suggested that attempts to expedite services or reduce unnecessary utilisation of diagnostic tests to reduce LOS may be worthwhile in reducing overall costs.

In terms of outpatients costs, physician services contributed to the bulk of the total expenditure in our study, and this was understandable since the growth in the number of physicians and specialists have increased over the years to meet with higher patient demands [32]. In addition, the introduction of new medical technologies and prescription drugs have also shown significant association with physician cost growth because consumers generally require physician visits to obtain diagnostic tests and prescriptions [32]. Because physicians are central to the healthcare system, efforts to contain physician spending reverberate through all healthcare services, especially with DM being a chronic condition requiring continuous follow-ups.

Our results from the regression analyses have generally confirmed what might have been expected based on the epidemiologic evidence in the literature [11, 20, 33–35], that microvascular and macrovascular complications tend to increase the cost of care. On the contrary, comorbidities such as hypertension and dyslipidaemia did not have an association with overall cost. This result is surprising since cost-effectiveness and medication adherence studies [36–39] have reported that achieving therapeutic clinical parameters would lead to an increase in cost of care albeit increasing the quality-adjusted life years (QALY). A possible explanation could be that hypertension and dyslipidaemia may have been controlled or at a steady state that did not require treatment, resulting in no costs incurred.

In our study, patients with sub-optimal and poor disease control had lower overall costs. This may be due to underutilisation of healthcare services compared to those with good disease control. The importance of managing DM to prevent or delay complications requires effort [40] and good control of DM results in long-term cost savings due to fewer complications [41]. Furthermore, The use of insulin only or both insulin and oral antidiabetic medications were found to be associated with higher costs. Consistent with other studies, the most expensive component of total outpatient costs after physician costs were medications [24, 25, 29, 42]. This rise in cost indicated a growth in the consumption of prescription medications, which may be due to increase adherence to medications. Evidence has shown that better adherence results in better healthcare outcomes and reduces the need for physician visits [43, 44], and lead to a net decrease in overall healthcare cost.

As a prevalence-based cost-of-illness study, the strength of this study was that all DM cases were included from a specified year, regardless of whether or not they were diagnosed before or during that year. This breadth allows for analysis of patients at various stages of the illness, since different severities of DM may be associated with different costs. However, there were several limitations in this study. First, data was drawn from a healthcare database, hence relied on the accuracy and completeness of the records. The NHG CDMS has, however, been used in several studies and is recognised for providing well-validated and comprehensive data [14, 45]. Second, patients with undiagnosed diabetes as well as indirect/intangible costs and out-of-pocket expenses were not included, which may contribute to an underestimation of the true cost of diabetes. Lastly, the study population was relatively small and limited to the public healthcare sector in Singapore. Future studies may consider these shortcomings to further assess different aspects of diabetes costs.

## Conclusion

This study provided a comprehensive cost analysis of expenditures incurred in the treatment of DM in Singapore. The results indicated that both medications and DM complications were strong determinants of costs. With projected increase in diabetes prevalence coupled with obesity and growing need for medical treatment in Singapore, diabetes will continue to be a heavy burden on health budgets. Therefore, evidence on the economic burden related to diabetes-related complication and its drives are indispensable for a health-system reform that seeks to minimise the long-term economic burden of this growing epidemic.
